# Reconstruction of chronic Achilles tendon ruptures using a doubled peroneus longus tendon free graft and interference screw fixation: a technical note

**DOI:** 10.1186/s13018-026-07162-y

**Published:** 2026-09-01

**Authors:** Nicola Maffulli, Cristiano Benelli, Niccolò Cacciuni, Vito Caiolo, Filippo Migliorini, Federico Morelli

**Affiliations:** 1 Orthopaedic Unit, Sant ’Andrea Hospital, University of Rome La Sapienza, Rome, Italy; 2 Department of Trauma and Reconstructive Surgery, University Hospital Halle, Martin-Luther University Halle-Wittenberg, Ernst-Grube-Street 40, 06097 Halle (Saale), Germany; 3 Department of Life Sciences, Health, and Health Professions, Link Campus University, 00165 Rome, Italy; 4 Department of Orthopaedic and Trauma Surgery, Eifelklinik St. Brigida, 52152 Simmerath, Germany

**Keywords:** Chronic Achilles tendon rupture, Peroneus longus autograft, Free graft, Interference screw, Mini-open reconstruction

## Abstract

**Background:**

The surgical management of chronic Achilles tendon ruptures (CATR) with significant tissue defects (Myerson Type II/III) presents a clinical challenge. Traditional reconstructive methods relying on free hamstring autografts or flexor hallucis longus transfers are often limited by donor-site morbidity, infection risks, or are unavailable in patients with a history of anterior cruciate ligament (ACL) reconstruction. This technical note describes a refined, tissue-preserving surgical technique utilising a doubled free autologous peroneus longus (PL) tendon graft.

**Methods:**

Under regional anaesthesia and thigh tourniquet control, a targeted 4 cm mini-open lateral incision is performed. The PL tendon is identified, harvested proximally using a tendon stripper, and a concomitant distal side-to-side tenodesis of the PL to the PB is performed to minimise eversion weakness. On the back table, the free PL autograft is doubled to achieve a 10 mm diameter construct. A calcaneal tunnel is drilled using progressive reaming. The free graft is routed through the proximal Achilles stump, passed subcutaneously, and drawn through the calcaneus. Final rigid stabilisation is secured with a 10 × 30 mm Bio-Interference Screw (Arthrex®) with the ankle held in maximum forced equinus to ensure optimal tensioning. Postoperatively, a 4-phase accelerated protocol begins with 3 weeks in a short-leg fibreglass cast under protected weight-bearing.

**Results/technical advantages:**

This technique achieves reliable primary mechanical stability, allowing for early protected loading while minimising soft-tissue stripping in the hypovascular distal Achilles watershed area. The free PL autograft provides an adequate structural match, and complements the translational framework of the Soft Tissue Healing Diamond (ST-Diamond) concept. Furthermore, this approach serves as a potential alternative for middle-aged patients who have a history of prior ACL reconstruction using hamstring autografts, avoiding contralateral donor-site morbidity or the use of allografts.

**Conclusion:**

The described mini-open free peroneus longus autograft reconstruction with interference screw fixation represents a cautious, reproducible, and biologically sound salvage option for large chronic Achilles tendon defects, balancing mechanical stability with minimal surgical morbidity.

## Introduction

Chronic Achilles tendon ruptures (CATR), defined as injuries diagnosed 4–6 weeks post-trauma, lead to triceps surae atrophy and marked loss of plantarflexion power [1–3]. Surgical management is guided by the post-debridement defect size, though the available guidelines are mainly based on expert consensus [4–6]. While defects < 2 cm may allow primary repair, gaps of 2–6 cm require reconstructive strategies [5–8]. Historically, free semitendinosus and gracilis autografts were commonly used to bridge this gap, but recent evidence highlights high complication rates (up to 24%), dominated by deep infections and skin necrosis [9, 10]. Furthermore, these reconstructions shorten the Achilles moment arm (mean reduction: 6.6 mm), potentially impairing long-term torque capacity [11, 12]. Local tendon transfer provides vascularized, biologically active tissue [1, 8]. Although flexor hallucis longus (FHL) transfer is frequently employed, it sacrifices the flexion strength of the hallux (up to 60%), and risks tibial nerve irritation [1, 4, 13]. The peroneus brevis (PB) tendon has been described as a local transfer option, with reliable results [14, 15]. In this landscape, the use of the ipsilateral peroneus longus (PL) tendon as a free graft could represent a suitable solution. Biomechanically, the PL tendon offers a significantly higher load-to-failure and greater graft diameter than both the FHL and the peroneus brevis, providing an excellent structural match to restore plantarflexion without compromising the function of the deep posterior compartment [14–16]. Furthermore, the substantial length available using the PL graft may facilitate optimal reconstruction of larger gaps; the concomitant distal tenodesis between the distal stump of the tendon of PL with the tendon of PB successfully prevents eversion weakness. We propose a minimally invasive reconstruction technique for CATR using a free ipsilateral peroneus longus tendon graft to restore the continuity of the gastrosoleus—Achilles tendon—calcaneus complex.

## Surgical technique

### Anaesthesia and positioning

The procedure is performed under regional anaesthesia (popliteal sciatic nerve block) with the patient prone and both feet hanging over the edge of the operating table. A pneumatic thigh tourniquet is applied to the upper thigh and inflated to 300 mmHg after limb exsanguination. Routine antibiotic prophylaxis is administered before the anaesthetic procedures are started.

### Graft harvest and preparation

**Fig. 1:**
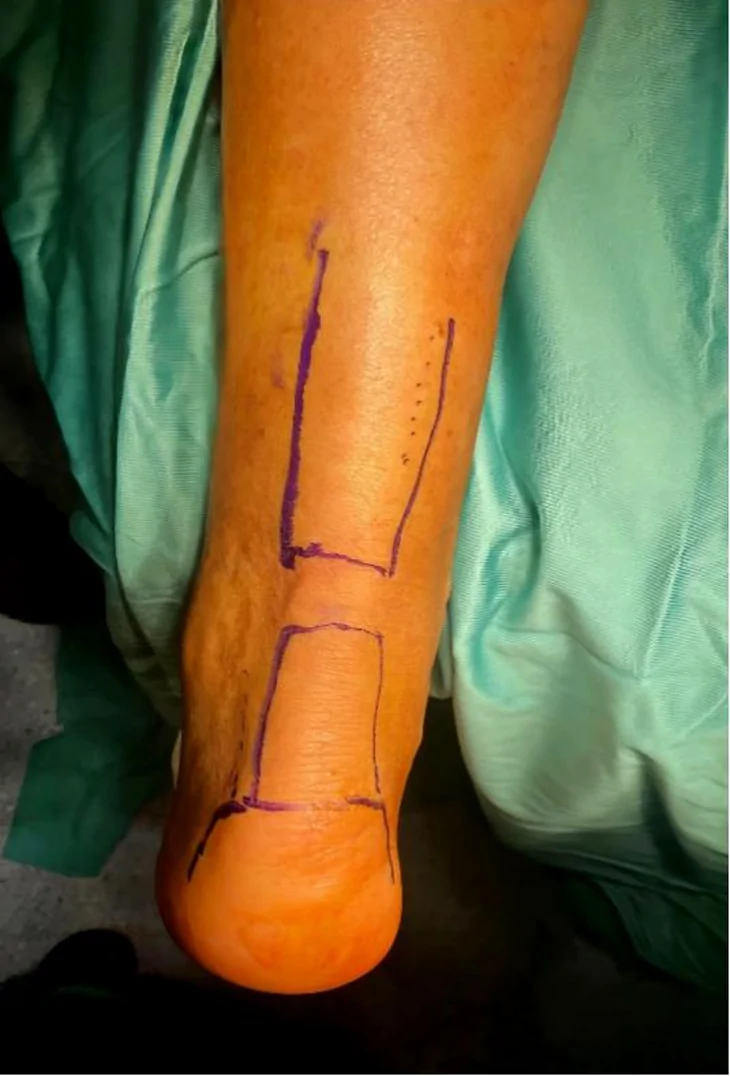
Surgical markings and anatomical landmarks. Preoperative clinical planning demonstrating the palpable Achilles tendon defect, the proximal and distal stumps, and the calculated pathway for the lateral incision, placed 18 mm medial to the expected course of the sural nerve

**Fig. 2:**
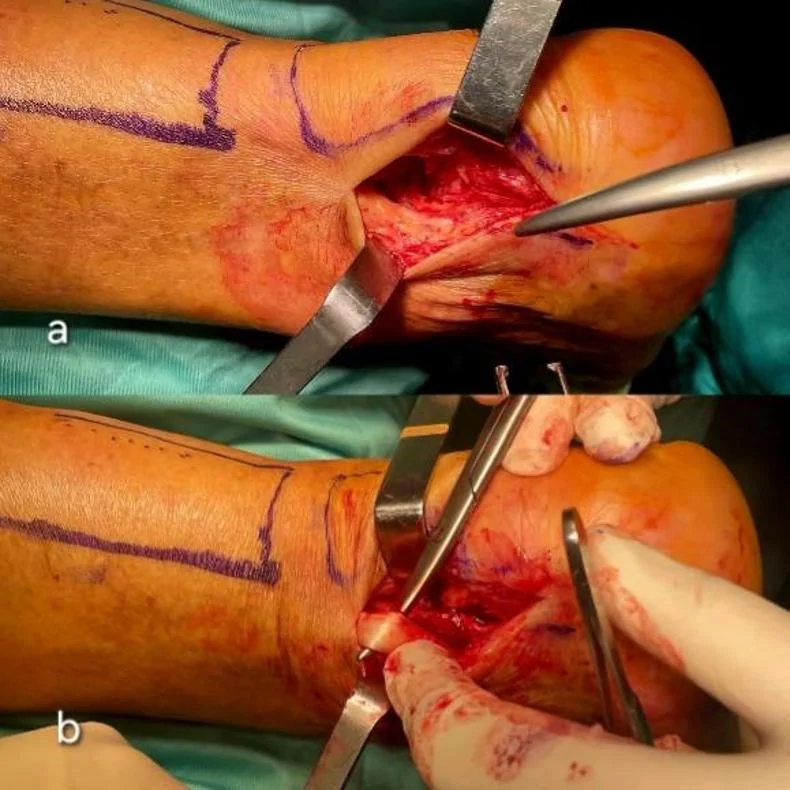
Isolation and exposure of the peroneal tendons. **a** Incision of the peroneal retinaculum and sheath. **b** Identification and safe isolation of the superficial peroneus longus (PL) and the more deeply situated peroneus brevis (PB) within the lateral compartment

**Fig. 3:**
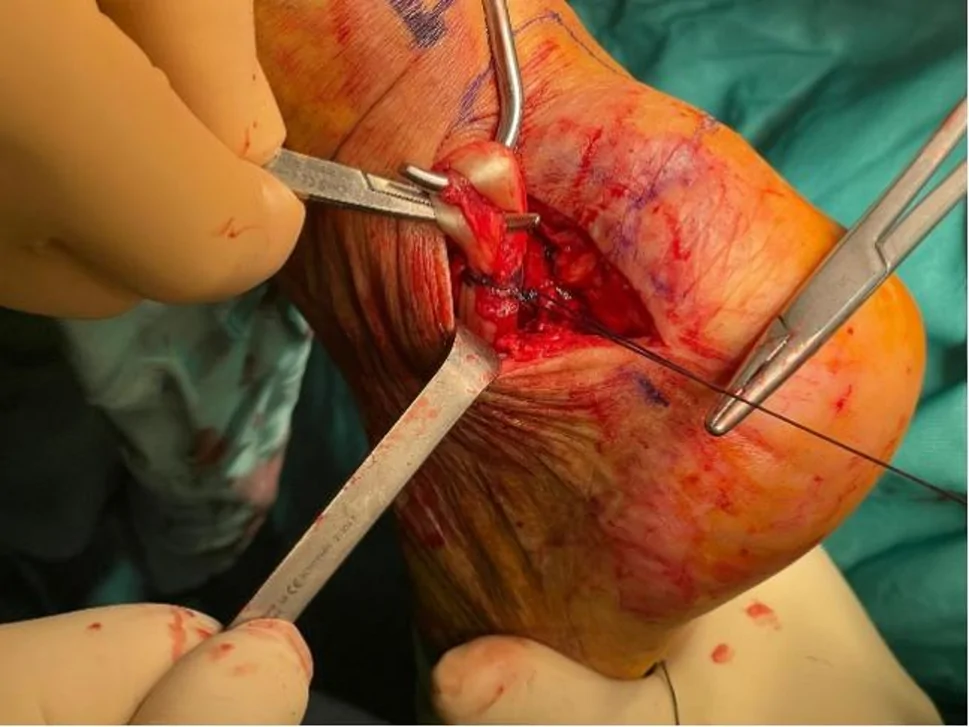
Distal side-to-side peroneal tenodesis. Intraoperative view of the side-to-side tenodesis performed between the PL and PB tendons approximately 2 cm proximal to the tip of the lateral malleolus using high-strength Vicryl® 2 (Ethicon) sutures

**Fig. 4:**
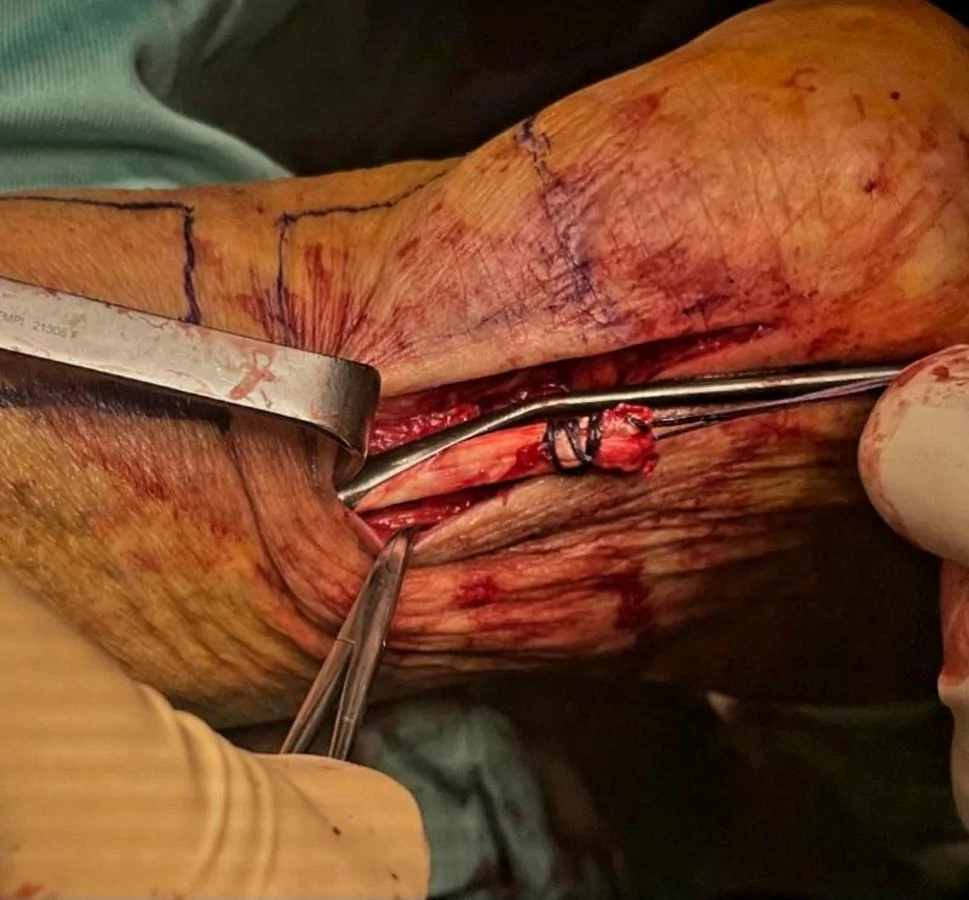
Free graft harvest. Transsection of the PL tendon just proximal to the tenodesis site and subsequent proximal harvesting up to the musculotendinous junction using a closed-ended tendon stripper

**Fig. 5:**
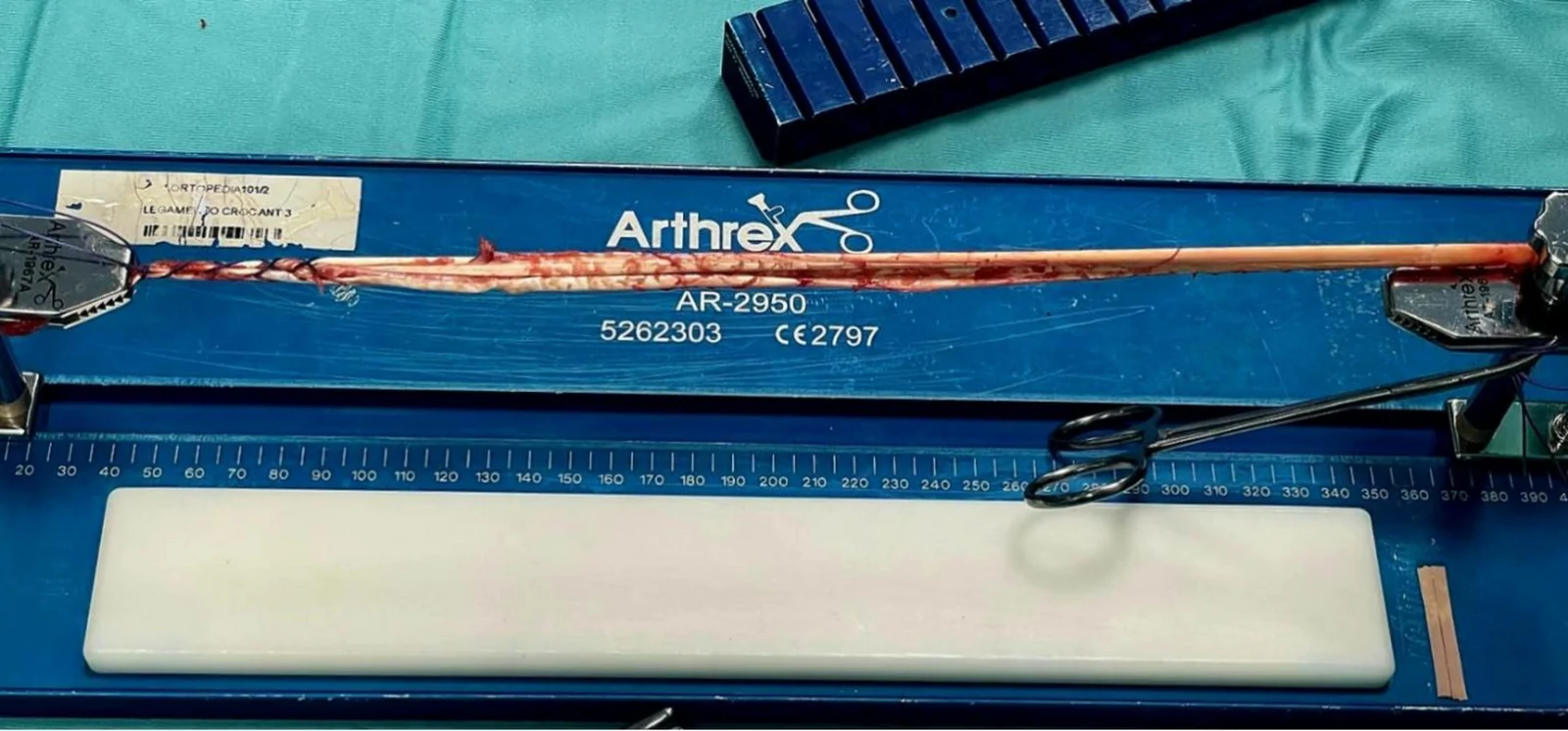
Free graft preparation on the back table. The harvested free PL autograft, cleared of muscular fibres, doubled to reach a final structural diameter of 10 mm, and whip-stitched at both ends using a Bunnell technique with high-strength Vicryl® 2 (Ethicon)

After routine skin preparation, sterile drapes are applied. The anatomical markings are defined: the palpable Achilles tendon defect, the distal and proximal tendon stumps, and the superior postero-lateral corner of the calcaneus (Fig. 1). A 4 cm lateral longitudinal incision is performed proximally to the lateral malleolus, taking care to stay close to the lateral border of the Achilles tendon and thus prevent damage to the sural nerve, which lies 18 mm lateral to the Achilles tendon insertion (Fig. 2a). The peroneal retinaculum and sheath are incised, identifying the more superficial peroneus longus (PL) and the deeper peroneus brevis (PB) (Fig. 2b). The PL tendon is isolated, and a distal tenodesis of the PL to the PB is performed approximately 2 cm proximally to the lateral malleolus using high-strength Vicryl® 2 (Ethicon) suture in a side-to-side fashion to prevent first ray instability and maintain eversion power (Fig. 3). Just proximally to the tenodesis site, the PL tendon is transected, secured with a Vicryl® 2/0 (Ethicon) stay-suture, and harvested proximally using a closed-ended tendon stripper up to the musculotendinous junction (Fig. 4). The PL graft is cleared of muscular fibers and measured. Each end of the PL graft is whip-stitched using the Bunnell technique with high-strength Vicryl® 2 (Ethicon).When doubled, the final diameter can reach 10 mm (Fig. 5).

### Achilles preparation and tunnel drilling

**Fig. 6:**
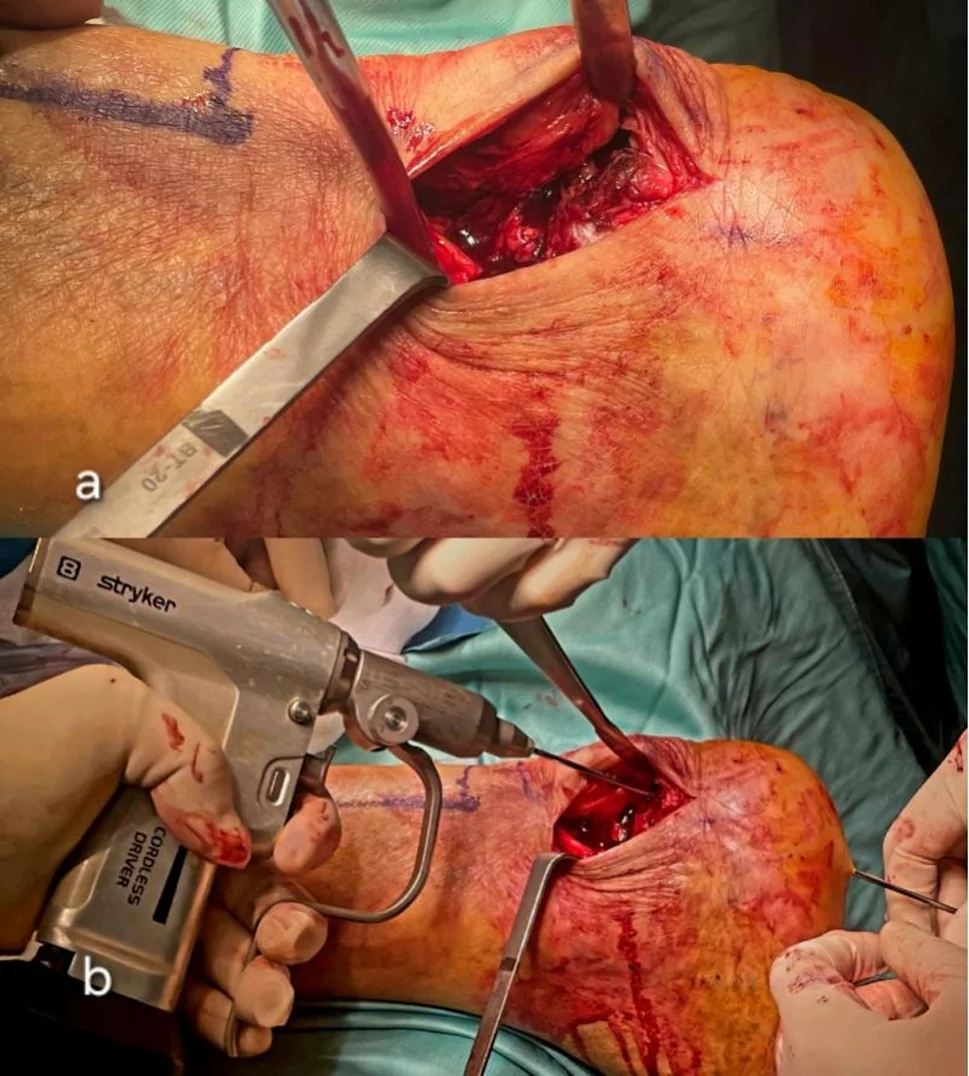
Calcaneal preparation and guide pin placement. **a** Identification and exposure of the superolateral angle of the calcaneus, followed by a limited postero-superior osteotomy performed with an oscillating saw to remove impinging bony spurs at the Haglund’s deformity area, without compromising the distal Achilles insertion. **b** Insertion of a guide Beath pin over the prepared postero-superior calcaneal footprint, inclined at approximately 45° and advanced in a proximal-to-distal (dorsal-to-plantar) direction

**Fig. 7:**
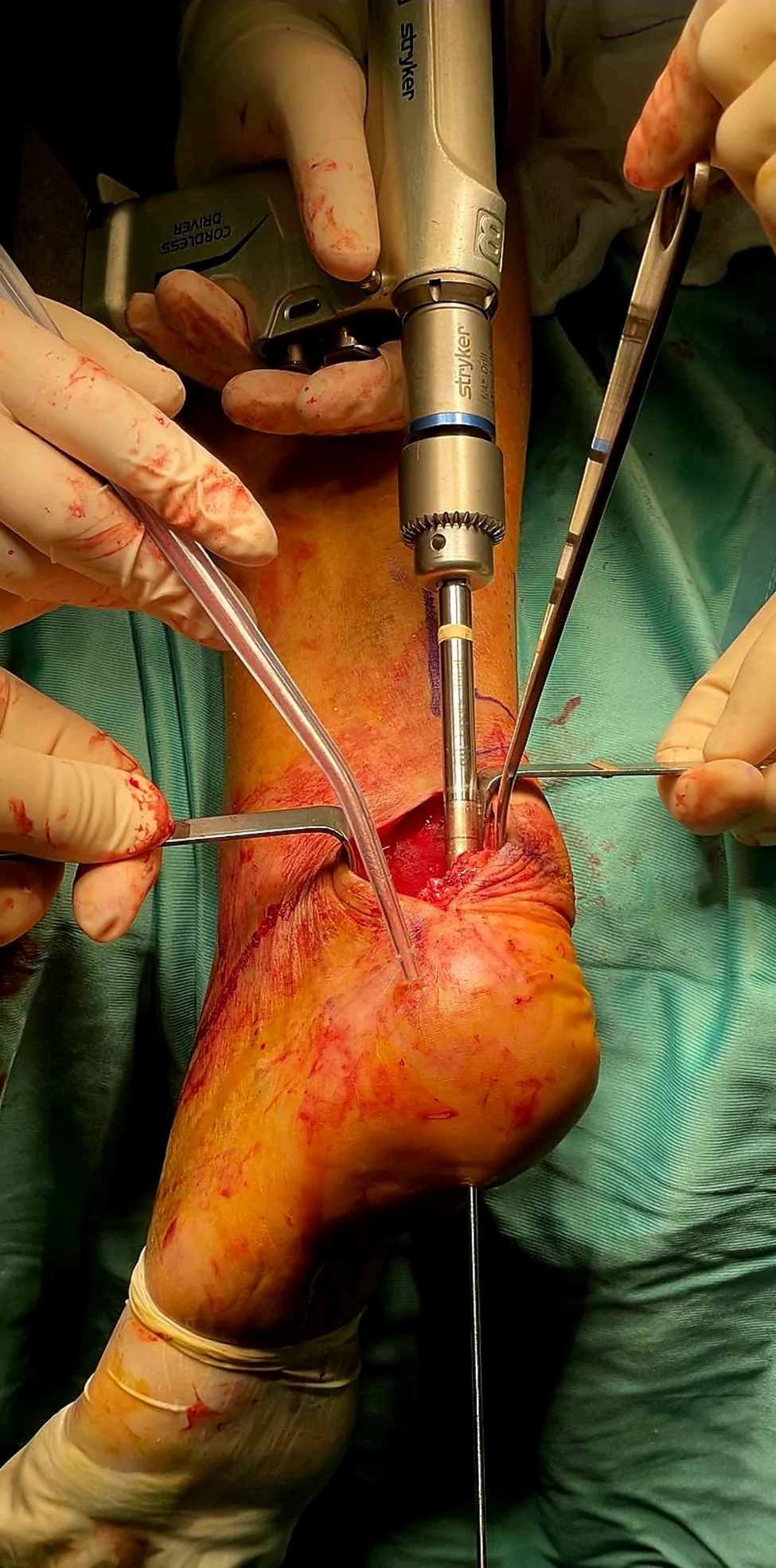
Calcaneal tunnel creation. Drilling of the calcaneal tunnel over a Beath pin inclined at approximately 45° to the horizontal, advanced from dorsal to plantar using progressive cannulated drill bits to match the doubled graft diameter

The Kager’s space and the postero-superior corner of the calcaneus are exposed distally, allowing to follow the course of the Achilles tendon up to the postero–superior corner of the calcaneum. An osteotomy is performed using an oscillating saw to expose the insertion of the Achilles tendon, paying attention to remove residual bony spurs which could impinge against the reconstructed Achilles tendon (Fig. 6a). This is a limited osteotomy of the postero-superior aspect of the calcaneum, without detachment of the insertion of the Achilles tendon, which lies below the lower margin of the osteotomy. A Beath pin inclined at about 45° to the horizontal, is inserted from dorsal to plantar through the posterior aspect of the calcaneus, as close at possible to the area of insertion of the Achilles tendon on the calcaneus (Fig. 6b). The calcaneal tunnel is then established using cannulated drill bits with progressively increasing diameters, reaching a final size to match the doubled PL graft diameter (Fig. 7).

### Graft passage and fixation

**Fig. 8:**
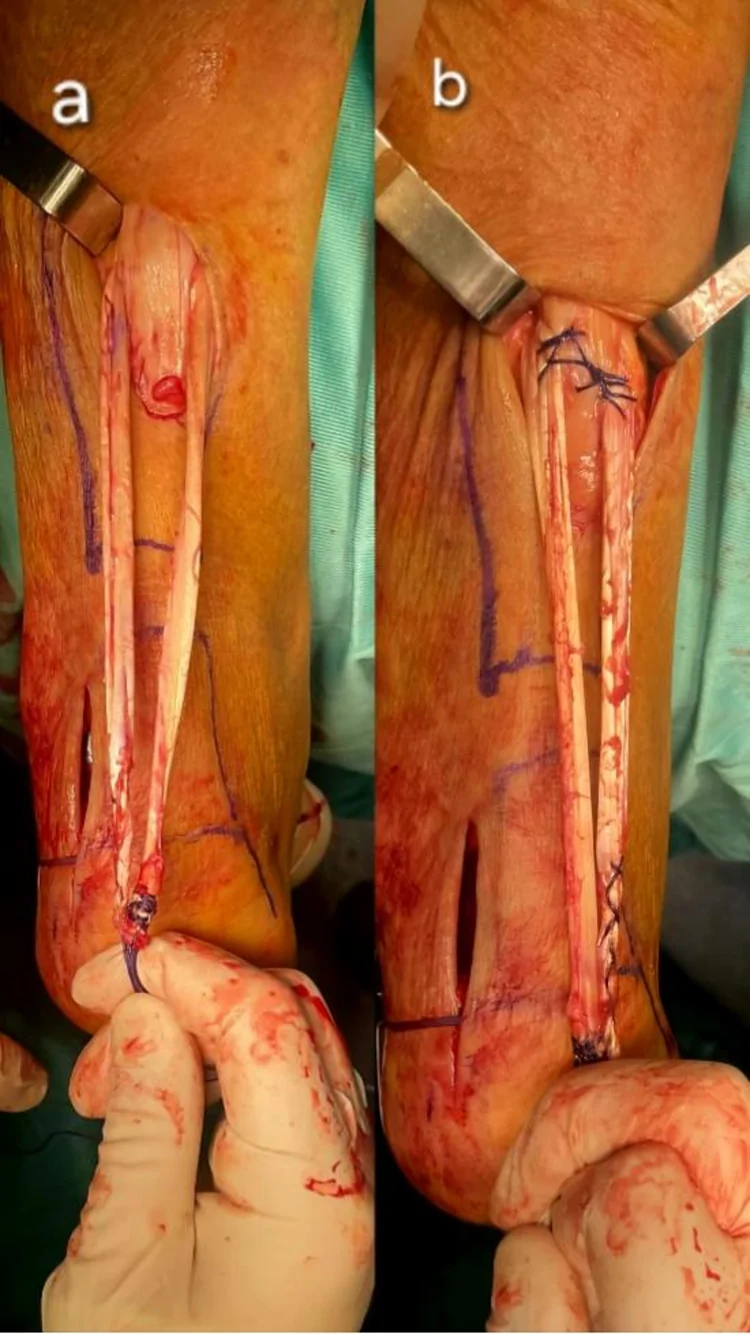
Proximal graft passage and paratenon management. **a** Introduction of the doubled free PL graft through the proximal Achilles tendon stump via a medial incision. **b** Secure direct closure of the paratenon and entry points using interrupted Vicryl® 2 (Ethicon) sutures

**Fig. 9:**
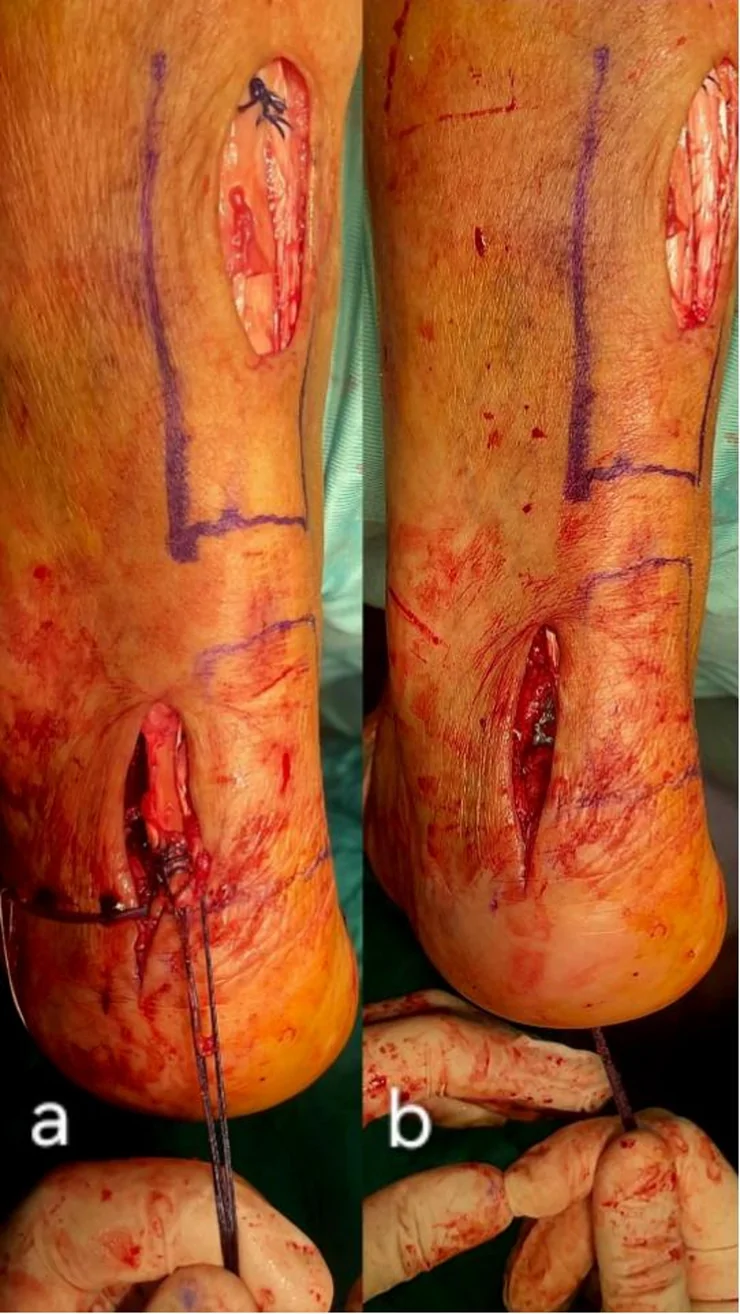
Subcutaneous graft routing and suture retrieval. **a** Subcutaneous translation of the graft ends anterior to the native Achilles tendon to successfully exit through the initial lateral incision site. **b** Introduction of a suture passer through the calcaneal tunnel to retrieve the graft stay-sutures, smoothly advancing the construct distally

A medial incision is made over the proximal Achilles tendon stump. Blunt dissection is used to expose the stump after incision and gentle retraction of the paratenon. The PL graft is passed horizontally through the proximal Achilles tendon stump, and the entry points sutured with Vicryl® 2 (Ethicon) (Fig. 8a, b). The graft ends are then passed deep (i.e. anterior) to the Achilles tendon to exit through the distal lateral peri-Achilles incision (Fig. 9a). A suture passer is introduced through the calcaneal tunnel to retrieve the stay-sutures, allowing the graft to be smoothly advanced through the calcaneus (Fig. 9b).

### Final fixation and closure

**Fig. 10:**
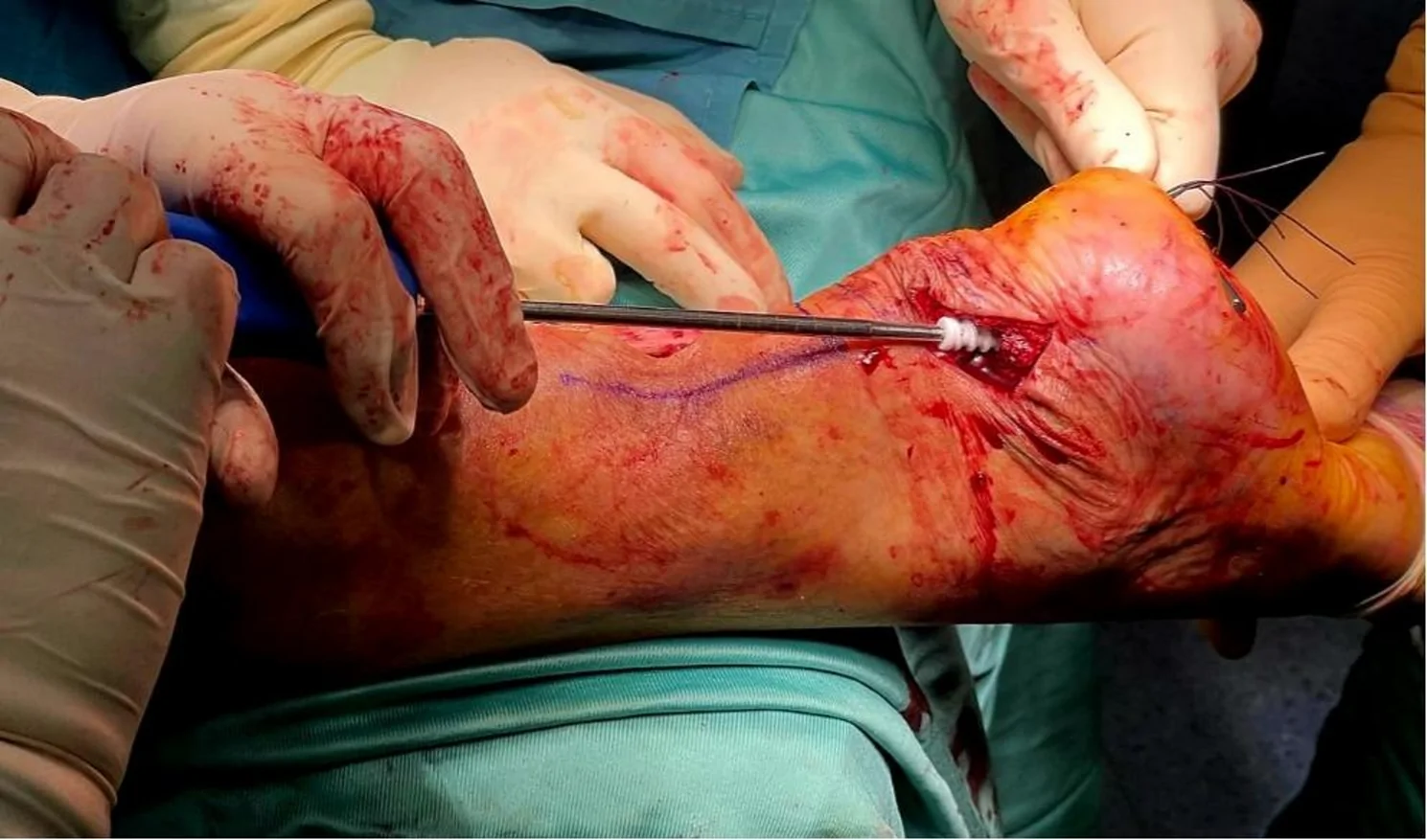
Final interference screw fixation. Final rigid stabilisation was achieved by inserting a 10 × 30 mm Bio-Interference Screw (Arthrex®) in a dorsal-to-plantar direction through the posterior calcaneal tunnel while maintaining the ankle in maximum forced equinus

Final stabilization is achieved by inserting a 10 × 30 mm interference screw (Bio-Interference Screw; Arthrex®, Naples, FL, USA) using a guide wire, in a dorsal to plantar direction through the calcaneal tunnel, while maintaining the ankle in maximum equinus to ensure optimal tensioning of the graft (Fig. 10). After thorough irrigation with normal saline, the deep tissues are sutured with vicryl, and the skin is closed using interrupted Monosyn® (B. Braun) 3-0 sutures. A sterile dressing and a short-leg fibreglass cast in equinus are applied.

### Postoperative management

Postoperative rehabilitation follows a structured, “slowed-down” accelerated protocol designed to protect the reconstruction, preserve the tension of the construct, and prevent long-term elongation. This protocol qualifies as “accelerated” as it allows immediate postoperative weight-bearing, which prevents triceps surae atrophy. Conversely, it is deliberately “slowed-down” by strictly prohibiting passive dorsiflexion and stretching until week 10, thereby safeguarding the healing bone-graft-anchor interface from excessive tensile forces and mitigating the risk of structural lengthening [17]. During the first 2 postoperative weeks, the ankle is immobilised in a below-knee synthetic cast in maximum equinus. Immediate full weight-bearing on the metatarsal heads using elbow crutches is permitted as tolerated. Active toe movement, knee range of motion, straight leg raises, and isometric calf contractions are initiated immediately to prevent muscle atrophy. At week 2, the cast is replaced by a removable rigid boot with 5 heel wedges to maintain maximum plantarflexion. Full weight-bearing in the boot is continued, and supervised physiotherapy begins, focusing exclusively on active plantarflexion, inversion, and eversion. Passive dorsiflexion and stretching are strictly prohibited. Crutches are typically discarded by week 4. The distinctive “slowed-down” phase occurs between weeks 6 and 12. Concentric gastro-soleus exercises and resisted eversion/inversion training are introduced after week 6. At week 10, the boot is progressively discontinued over 2 weeks (removed for 1–4 h daily). When out of the boot, patients bear weight using a 15 mm heel lift. Advanced rehabilitation commences after month 4, with eccentric calf exercises introduced at week 16. Plyometric training is permitted at month 5. Patients return to regular activities and low-impact sports after the fifth month, pending clinical clearance demonstrating satisfactory single-leg heel-rise capacity, symmetric calf circumference, and functional stability.

## Discussion

Reconstruction of CATR remains a surgical challenge, particularly when large tendon gaps are present [4, 5]. Various techniques have been described, and the mini-open free PL graft reconstruction with interference screw fixation may offer several advantages over traditional methods.

### Surgical efficiency and reduced morbidity

One of the primary strengths of our technique is the reduced surgical time and complexity. Unlike hamstring autografts, which require a separate harvest site over the pes anserinus or in the popliteal fossa and may carry a risk of donor-site morbidity [9], the PL graft is harvested through the same lateral approach used to expose the distal Achilles tendon and undertake the osteotomy of the posterosuperior corner of the calcaneus. This proximity minimises the number of distinct skin incisions, reducing the surgical footprint. The use of a PL graft may offer a critical advantage, considering that hamstring autografts might not always be available, as they may have been utilised in previous surgical procedures. While anterior cruciate ligament (ACL) tears show a high incidence among adolescents and young adults under 25 years [18], CATR characteristically peak in middle-aged individuals between 30 and 59 years [2, 14]. Given this demographic reality, middle-aged patients presenting with a chronic Achilles tendon rupture may have a history of prior ACL reconstruction using a hamstring autograft [18]. In this population, traditional salvage procedures for Achilles reconstruction that rely on free semitendinosus or gracilis autografts are simply not viable because of the prior donor-site harvest. Using a free autologous PL tendon graft eliminates the financial burden of expensive allografts and the necessity for additional donor-site morbidity in an uninjured limb.

### Functional preservation

A key rationale for selecting the PL autograft over the FHL transfer is the preservation of deep posterior compartment mechanics and sagittal gait parameters. The FHL is the primary flexor of the hallux, and contributes significantly to the push-off phase of gait; its sacrifice can lead to a 60% reduction in hallux flexion strength and potential balance impairments [4, 13]. While the PL is a major foot evertor and a plantarflexor of the first ray, its harvest does not cause significant morbidity when combined with a concomitant distal tenodesis with PB [19]. Nevertheless, a balanced clinical evaluation requires acknowledging potential donor-site limitations. Harvesting the PL autograft introduces theoretical concerns regarding ankle eversion instability and first ray dysfunction, given the muscle’s role in stabilizing the medial longitudinal arch. However, executing a meticulous distal side-to-side tenodesis of the PL to the peroneus brevis effectively mitigates these risks, allowing the peroneus brevis to functionally compensate for the loss of the proximal PL motor unit without major long-term sequelae.

### Biomechanical superiority and early loading

In our technique, the PL graft is doubled, reaching a diameter up to 10 mm, and is secured with a proximal-to-distal calcaneal interference screw, which provides a very high initial stability [7, 13]. This immediate mechanical rigidity satisfies the translational framework of the ‘Soft Tissue Healing Diamond’ (ST-Diamond) concept: optimising the local mechanical environment through early controlled strain is a crucial prerequisite to drive progenitor cell differentiation and successful tendon regeneration [20]. By achieving a dependable bone-tendon interface, early protected weight-bearing protocols can be implemented. Construct robustness is vital when applying rehabilitation strategies based on immediate post-operative weight bearing, preventing muscle atrophy and tendon elongation, which are often associated with prolonged cast immobilisation or overly aggressive immediate physical therapy [11, 17].

### Wound safety

The “watershed” area of the distal Achilles tendon is notoriously vulnerable to wound healing disorders, skin edge necrosis, and devastating deep infections, given its poor baseline vascularity [9]. A primary advantage of our targeted mini-open technique is the avoidance of extensive surgical wounds, thus minimising soft-tissue insult, limiting the disruption of the residual paratenon, and preserving the surrounding blood supply. By performing a deep graft passage and confining the calcaneal fixation to a precise posterior portal, we effectively protect the fragile overlying skin envelope from excessive tension. This strategy aligns with the latest paradigms in minimally invasive and endoscopic-assisted footprint reconstructions, which have consistently demonstrated near 0% wound complication rates by respecting the local angiosome and accelerating early soft-tissue sealing [21].

### Limitations

We acknowledge that this article presents limitations. First, long-term clinical follow-up data regarding functional outcomes, donor-site morbidity, and potential graft elongation are not yet available. Second, this mini-open approach entails an inherent learning curve, as surgeons may not be familiar with the anatomical dissection necessary to harvest the tendon of peroneus longus. Lastly, despite the extensive anatomical safeguards described to preserve the surrounding structures, a theoretical risk of sural nerve irritation or injury remains given the proximity of the lateral surgical access.

## Conclusions

The technique described provides a robust and biologically sound reconstruction in patients using CATR using a free ipsilateral PL autograft. Using a mini-open approach, performing a distal peroneus longus-to-peroneus brevis tenodesis, and employing interference screw fixation, this method ensures high “time-zero” stability, allows for protected active postoperative rehabilitation, and significantly minimises the risk of wound complications or sural nerve injury. Furthermore, it offers a powerful alternative to hamstring or flexor hallucis longus transfers, effectively restoring plantarflexion torque and preserving donor-site morbidity without compromising long-term eversion power. This technique could bridge the gap between traditional open reconstructions and purely endoscopic methods, offering a reproducible and effective solution for chronic Achilles tendon ruptures.

## Data Availability

The datasets generated during and/or analysed during the current study are available throughout the manuscript.

## Declarations

### Use of AI

Language editing was performed using Grammarly (Superhuman Platform Inc., San Francisco, CA, USA).
